# 512-Channel and 13-Region Simultaneous Recordings Coupled with Optogenetic Manipulation in Freely Behaving Mice

**DOI:** 10.3389/fnsys.2016.00048

**Published:** 2016-06-14

**Authors:** Kun Xie, Grace E. Fox, Jun Liu, Joe Z. Tsien

**Affiliations:** ^1^Brain and Behavior Discovery Institute–Department of Neurology, Medical College of Georgia, Augusta University, AugustaGA, USA; ^2^The Brain Decoding Center, Banna Biomedical Research Institute, Yunnan Academy of Science and TechnologyYunnan, China

**Keywords:** large-scale recordings, optogenetics, learning and memory, earthquakes, fear conditioning, functional connectivity, BRAIN project, neural code

## Abstract

The development of technologies capable of recording both single-unit activity and local field potentials (LFPs) over a wide range of brain circuits in freely behaving animals is the key to constructing brain activity maps. Although mice are the most popular mammalian genetic model, *in vivo* neural recording has been traditionally limited to smaller channel count and fewer brain structures because of the mouse’s small size and thin skull. Here, we describe a 512-channel tetrode system that allows us to record simultaneously over a dozen cortical and subcortical structures in behaving mice. This new technique offers two major advantages – namely, the ultra-low cost and the do-it-yourself flexibility for targeting any combination of many brain areas. We show the successful recordings of both single units and LFPs from 13 distinct neural circuits of the mouse brain, including subregions of the anterior cingulate cortices, retrosplenial cortices, somatosensory cortices, secondary auditory cortex, hippocampal CA1, dentate gyrus, subiculum, lateral entorhinal cortex, perirhinal cortex, and prelimbic cortex. This 512-channel system can also be combined with *Cre*-lox neurogenetics and optogenetics to further examine interactions between genes, cell types, and circuit dynamics across a wide range of brain structures. Finally, we demonstrate that complex stimuli – such as an earthquake and fear-inducing foot-shock – trigger firing changes in all of the 13 brain regions recorded, supporting the notion that neural code is highly distributed. In addition, we show that localized optogenetic manipulation in any given brain region could disrupt network oscillations and caused changes in single-unit firing patterns in a brain-wide manner, thereby raising the cautionary note of the interpretation of optogenetically manipulated behaviors.

## Introduction

Understanding how the brain generates perception, memory, and behavior requires large-scale mapping of brain activity patterns (Buzsáki, 2004; Tsien, 2007; Alivisatos et al., 2012; Mitra, 2013; Tsien et al., 2013), new analytical tools (Oşan et al., 2007, 2011; Donner and Siegel, 2011; Stevenson and Kording, 2011; Cunningham and Yu, 2014) as well as novel conceptual framework (Tsien, 2015, 2016a; Schneidman, 2016). In particular, identification of dynamic network patterns is crucial for bridging the knowledge gap between genes and cognitions (Grant et al., 1992; Silva et al., 1992; McHugh et al., 1996; Tsien et al., 1996, 2013; Tang et al., 1999; Feng et al., 2001; Cui et al., 2004; Dragoi and Buzsáki, 2006; Takehara-Nishiuchi and McNaughton, 2008; Wang et al., 2011; Zhang et al., 2013; Randlett et al., 2015; Gozzi and Schwarz, 2016; Tsien, 2016b). To date, microelectrodes remain the gold-standard method with high temporal resolution and the ability to measure excitatory and inhibitory neuronal activities at all depths throughout the brain. At the same time, they also allow researchers to measure LFP, thereby enabling additional analysis of dynamic interactions between individual neurons and local networks (Adrian and Moruzzi, 1939; Ling and Gerard, 1949; Lorente de Nó, 1949; Mountcastle, 1957; Hubel and Wiesel, 1962; Hamill et al., 1981; Pinault, 1996; Klausberger and Somogyi, 2008; Uhlhaas et al., 2010; Donner and Siegel, 2011).

Studies based on one or few electrodes typically require the activity of single units to be averaged over many trials in order to overcome firing variability and to define their response properties. However, the brain is unlikely to use this trial-averaging practice to achieve real-time encoding and representation of the world (Lin et al., 2006b; Stevenson and Kording, 2011). In recent years, large-scale ensemble neural recording technologies have been making rapid progress (Buzsáki, 2004; Lin et al., 2006a; Dzirasa et al., 2011; Fan et al., 2011; Berényi et al., 2014; Schwarz et al., 2014). Extensive evidence shows that recording a large number of neurons can help better the understanding of complex neural patterns associated with motor control (Georgopoulos et al., 1986), spatial navigations (Harris, 2005; Pastalkova et al., 2008; Takehara-Nishiuchi and McNaughton, 2008), and real-time fear memory traces (Lin et al., 2005; Chen et al., 2009; Tsien et al., 2013; Zhang et al., 2013).

Mice are the best known genetic organisms to offer various powerful molecular genetic tools for the study of genes, neural circuits, and behaviors (Tsien et al., 1996; Tang et al., 1999; Cui et al., 2004; Cao et al., 2008; Wang et al., 2011). In particular, *Cre*/loxP neurogenetics has opened a door to define and manipulate specific cell types and neural circuits in the brain (Tsien et al., 1996; Tsien, 2016b). Many Cre-driver mouse lines are now routinely combined with the opsin-based light-manipulation of neurons *in vivo* (Boyden et al., 2005; Deisseroth, 2011; Leão et al., 2012; Madisen et al., 2012; Tsien, 2016b). These genetic resources and tools make mice particularly attractive for large-scale mapping of brain activity patterns in both normal and diseased states. However, many electrode configurations used for the study of rats or primates are often unsuitable for mice because of their much smaller body size. Another challenge is that the mouse’s thin skull makes it difficult to affix the large microdrive and headstage for stable, chronic recordings. This has explained, in part, why the vast majority of electrodes used in freely behaving mice is still largely limited to 32 or fewer channels and to only one or two brain structures. Here, we describe a 512-channel tetrode system that can monitor neural activity from 13 different cortical and subcortical structures in freely behaving mice. We further show that this new system can be combined with multiple optical fibers for the *Cre*/loxP-mediated optogenetic probing of the dynamic interactions across different cell types and brain regions.

## Materials and Methods

### Ethics Statement

The animal protocol was approved by the Animal Use and Care Committee at Augusta University. All animals were maintained by the trained Animal Facility staff and an experienced veterinarian who conducted routine daily health surveillance. All animal handling and tissue preparation were performed in accordance with NIH guideline and the protocols approved by IAUCC committee at Augusta University.

### Configuration of the 512-Channel Headstage for Targeting 13 Cortical and Subcortical Structures

Thirteen different mouse brain structures were selected for simultaneous recordings in the present study; their names and stereotaxic coordinates are as follows: (1) S1 trunk region (S1Tr) of the somatosensory cortex (-1.6 mm AP, ± 1.75 mm ML); (2) S1 hind limb (S1HL) of the somatosensory cortex (-1.1 mm AP, ± 1.5 mm ML); (3) secondary auditory cortex, ventral portion (AuV, -1.94 mm AP, ± 4.75 mm ML); (4) hippocampal CA1 (-3.8 mm AP, ± 3.0 mm ML); (5) dentate gyrus (DG, -3.75 mm AP, ± 2.0 mm ML); (6, 7) Cg1 of the anterior cingulate cortex (+0.50 mm AP, ± 0.3 mm ML); (7) Cg2 of the anterior cingulate cortex (+0.50 mm AP, ± 0.6 mm ML); (8) subiculum (S, -3.08 mm AP, ± 1.5 mm ML); (9) lateral entorhinal cortex (LEnt, -3.80 mm AP); (10) agranular cortex (RSA) of the retrosplenial cortex (-2.3 mm AP, ± 0.6 mm ML); and (11) granular cortex (RSG) of the retrosplenial cortex (-2.3 mm AP, ± 0.3 mm ML); (12) perirhinal cortex (PRh, -3.80 mm AP); and (13) prelimbic cortex (PrL, +1.70 mm AP, ± 0.5 mm ML). All positions were measured with respect to the bregma point. The electrode positions on the microdrive headstage were pre-calibrated according to these brain-region coordinates, which were based on the Mouse Brain Atlas (Paxinos and Franklin, 2001).

Because some of these 13 brain regions are located next to each other, the 512-channel headstage was designed and made with seven separate polyimide-tube modules. Parts, tools, and suppliers for constructing the headstage are provided (see Supplementary Table S1). Within each module, segments of polyimide tubing (inner diameter 99.9 μm, outer diameter 167 μm, Polymicro Technologies, Phoenix, AZ, USA) were arranged in arrays that contained one bundle or multiple bundles, which allowed one brain site or multiple nearby sites to be targeted, respectively. The purpose of polyimide tubing was to make tetrode arrays into various pre-designed configurations and also to hold each tetrode in position. These seven modules were as follows: (1) LEnt/PRh-module; (2) RSG/RSA-module; (3) S1HL/S1Tr-module; (4) CA1/DG-module; (5) ACC (Cg1/Cg2)/PrL-module; (6) AuV-module; and (7) subiculum (S)-module. The size and arrangement of tetrode bundles within each module depended on the regions that were to be targeted.

To target the LEnt and PRh regions, the LEnt/PRh module consisted of three arrays of polyimide tubes (with each tube holding one tetrode). Eight tubes in a row were used for the PRh and two rows of eight tubes for targeting the LEnt. These three 1 × 8 tetrode rows were separated by empty rows of polyimide tubes with 1 × 8 dimensions. To target the RSA and RSG, the RSA/RSG-module consisted of two 1 × 8 bundle rows – one row targeting the RSA and the other for the RSG. A 1 × 8 row of polyimide tubes was used to spatially separate these two rows. The same design was also used for constructing module #3 for recordings from the S1TR and the S1HL.

For recordings in the CA1 and DG, we used the microdrive design so that electrodes can be lowered to the pyramidal cell or granule cell body layer. Individual polyimide modules for targeting CA1 and DG used a 4 × 4 tetrode arrangement and were secured to independently movable screw nuts on the base of a microdrive that was constructed from fiberglass. Because these sites are only one cell-layer thick, this hippocampal module contained no spatial separation by empty polyimide tubes.

To record in the Cg1, Cg2, and the PrL, we used a single module consisting of 3 × 14 polyimide-tubing array. A 1 × 8 tetrode row was used to target the Cg1 and another parallel row for the Cg2. These two tetrode rows were separated by one 1 × 8 row of empty polyimide tubing. We constructed the PrL tetrode bundle using two rows of four tetrodes separated by a row of empty tubing. The PrL tetrode bundle and Cg1/Cg2 bundles were separated by a distance of 0.32 mm (using two columns of empty polyimide tubes). The remaining two modules were used to target the S and AuV, respectively. The modules consist of an optical fiber (200 μm core, 037NA standard cladding multimode fiber from ThorLabs, Newton, NJ, USA) surrounded by a total of 12 polyimide tubes (eight filled with tetrodes, four empty). There was one 1 × 4 row above the optical fiber, one 1 × 4 row below the fiber, and one 1 × 4 row on each side of the fiber. One empty polyimide tube was positioned at the four corners to secure them. Because of the different depths for each region, the tetrodes were cut into corresponding lengths.

A cross-shaped planar fiberglass was made to secure four 128-channel connector pins (each consisting of four rows of 36 pin-connector arrays) on each arm by DIY CNC carving Machine (Zen Toolworks CNC DIY KIT, F8 version, ZEN Toolworks, Walnut Creek, CA, USA). This design also contained a small fiberglass base at the bottom to aid in affixing the entire headstage to the skull. In each arm, four 36 pin-connector arrays (A79026-001, Omnetics Connector Corporation, Minneapolis, MN, USA) were positioned and secured with epoxy (5 Minute Epoxy Gel, ITW Polymers Adhesives North America, Danvers, MA, USA) in a parallel manner. The individual headstages with fiberglass bases were attached to small copper bars with Loctite Super Glue (STAPLES, Framingham, MA, USA).

### Construction of Tetrodes for 512-Channel Recordings

To construct tetrodes, four wires (90% platinum, 10% iridium, 13 μm, California Fine Wire Company, Grover Beach, CA, USA) were twisted together using a manual turning device and soldered with a low-intensity heat source (variable temperature heat gun 8977020, Milwaukee, Brookfield, WI, USA) for 6 s. The impedances of the tetrodes were measured with an electrode impedance tester (Model IMP-1, Bak Electronics, Umatilla, FL, USA) to detect any faulty connections, and our tetrodes were typically between 0.7 and 1 MΩ. The insulation was removed by moving the tips of the free ends of the tetrodes over an open flame for approximately 3 s. A tetrode length of 7 or 9.5 cm was optimal so that each of the 13 sites could accurately be reached. The tetrodes were then placed into appropriate polyimide tubes. As previously mentioned, the modules that were to target the RSA and RSG, S1Tr and S1HL, Cg1, Cg2, and PrL, PRh, and LEnt were designed so that the bundles could be spatially separated with rows of empty polyimide tubes. This allowed for a larger recording area in each brain site of interest, as well as creating a distance between bundles recording from different brain sites.

Importantly, the recording ends of the tetrodes were cut differentially (Vannas spring scissors -3 mm cutting edge, Fine Science Tools, Foster City, CA, USA) so that multiple recording sites, located at different depths, could be reached with the same module. This ensures that only tetrodes, but not the surrounding polyimide tubes, were inserted into the brain tissue, thereby minimizing tissue damage. The following measurements were the tetrode lengths protruding from the polyimide tubes: RSA 4.5 mm, RSG 5 mm, Cg1 4.5 mm, Cg2 5 mm, PrL 5.5 mm, PRh 4.75 mm, and LEnt 4.9 mm and 5 mm (this maximized the area that could be recorded from this site). The tetrodes targeting the CA1 and DG were cut at lengths of 5 and 5.75 mm, respectively. A reference wire was then soldered to the final four pins on each end of the connector. Finally, the connector-pin array was coated with epoxy and, for recordings in the CA1 and the DG, the looped tetrodes were glued to the base of the microdrive. For the remaining arrays, the free portions of the tetrodes were tied together and would later be secured to the fiberglass base toward the end of the surgery.

### Construction of Optrodes and Light Stimulation Protocol

We selected two regions – namely, the subiculum and auditory cortex – for optogenetic manipulation in our 512-channel headstage. The optrodes were constructed in a square-shaped arrangement of 12 polyimide tubes (inner diameter 99.9 μm, outer diameter 167 μm, Polymicro Technologies, Phoenix, AZ, USA) on the four sides surrounding the optical fiber in the center. For optical fiber, cladding was removed from the two 200-μm core, 037NA standard, hard-cladding, multimode fiber (ThorLabs), and the optical fibers were placed 1 mm apart in a microdrive base. Eight tetrodes were threaded into separate polyimide tubes and placed adjacent to each optical fiber to create an optrode bundle with the tip of the optical fibers 600 μm above the recording tetrodes. Tetrodes were then inserted into eight of the tubes, leaving an empty tube at each corner of the square. This arrangement would allow the tetrodes to be reached effectively by the light stimulation from the fiber. After each tetrode was correctly positioned, the wires were glued to the polyimide tubes. The free ends of each tetrode were wrapped around the 36 pin-connector array and were individually soldered to their respective pins. The recording tips of the tetrodes were cut so that they would protrude past the fiber 300–500 μm.

Prior to stimulation, PM100D (ThorLabs) was used to measure the light intensity. Optical fibers were connected to a green laser (532 nm, diode-pumped solid-state, Shanghai Dream Laser Technology Co., Shanghai, China). Trains of 10-Hz stimulations (10 ms per pulse, 20 pulses per train) were delivered to the AuV or S in 10 trials using a PulsemasterA300. With 532 nm at 10 mW, it generated the intensity of ∼318 mW mm^-2^ at the 200-μm fiber tip, and ∼14 mW mm^-2^ at the recording sites. Optogenetic suppression of putative pyramidal cells was identified by measuring the reduction of the firing rate during and after light stimulation. We also compared waveforms before and after optical stimulation. Waveforms were judged to be identical if the correlation coefficient was measured to be higher than 0.9.

### Surgery for Implanting 512-Channel/13-Site Electrode Array

Wild-type B6BCA/J and CaMKII-Cre (T62)::Arch double transgenic mice were given *ad libitum* access to food and water in their home cages and were handled for several days prior to the surgery to reduce potential stress caused by human interaction. On the day of the surgery, a mouse was given an intraperitoneal injection of 60 mg/kg ketamine (Bedford Laboratories, Bedford, OH, USA) and 4 mg/kg Domitor (Pfizer, New York, NY, USA) prior to the surgery. The head of the mouse was secured in a stereotaxic apparatus, and an ocular lubricant was used to cover the eyes. The hair above the surgery sites was removed, and Betadine solution was applied to the surface of the scalp. An incision was then made along the midline of the skull. Hydrogen peroxide was placed onto the surface of the skull so that bregma could be visualized. The correct positions for implantation were then measured and marked. For each mouse, all electrode arrays were targeted to the same hemisphere. Four holes for screws (B002SG89S4, #00-90, 1/8 inches, Amazon, Seattle, WA, USA) were drilled on the opposing side of the skull and, subsequently, the screws were placed in these holes with reference wires being tightly wrapped around two of the head screws to secure them. Craniotomies for the tetrode arrays were then drilled, and the dura mater was removed. Vaseline was applied to the implantation sites after the electrodes were inserted. Because of the large number of recording sites and a surgery time lasting 5∼6 h, the sequence of the implantations was important to ensure optimal recording conditions as follows:

First, the module that was to record from the RSA and RSG was implanted at -2.3 mm AP, ± 0.5 mm ML, 0.8 mm DV. Using the manipulator arm of the stereotaxic device, these AP and ML measurements were made relative to bregma. The depths of all implantations, however, were in relation to the surface of the brain. Dental cement was subsequently used to secure the array. After the dental cement dried, the copper bars that held the arrays were removed from the fiberglass bases.

Second, the S1Tr and S1HL module was implanted at -1.6 mm AP, ± 1.75 mm ML and -1.1 mm AP, ± 1.5 mm ML, respectively, at a depth of 0.5 mm. Also important, in order to better achieve multi-site recording, this array was inserted laterally at a 45° angle. The array was then secured with dental cement.

Third, the ACC and PrL module was then targeted to +0.50 mm AP, ± 0.5 mm ML and +1.70 mm AP, ± 0.5 mm ML, respectively, at a depth of 3.5 mm and was fastened with dental cement.

Fourth, the S module was inserted at -3.08 mm AP, ± 1.5 mm ML, 1.25 mm DV. Dental cement was used to secure the array. The AuV module was implanted at -1.94 mm AP, ± 4.75 mm ML. This array was inserted at a 45° angle and a depth of 0.5 mm. Dental cement was applied to affix the array.

Fifth, the CA1 and the DG were targeted, and the modules were implanted at -1.94 mm AP, ± 4.75 mm ML 1.0 mm DV and -3.75 mm AP, ± 2.0 mm ML, 1.75 mm, respectively, at 45°. The microdrive was also fastened with dental cement.

Finally, the PRh and LEnt module was implanted at -3.80 mm AP, 0.4 mm DV at a 70° angle. Dental cement was used to secure this array and to firmly affix the remaining arrays. Following that, the cross-shaped planar fiberglass was secured with dental cement (Dental cement kit, pink opaque, Stoelting, Wood Dale, IL, USA). The tetrodes and connector pins from each electrode were then secured to this base. The reference wires from the connector-pin arrays were soldered such that there would be a continuous circuit between the ground wires from the head screws and those from the connector-pin arrays. Aluminum foil was used to surround the entire headstage to aid in protection and to reduce noise during recordings. The mouse was injected with 2.5 mg/kg Antisedan (Pfizer, New York City, NY, USA) and placed in its home cage. Importantly, the weight of the headstage was approximately 11.5 g; thus, helium balloons were used to offset the weight of this device. By using this technique, the mice were able to move freely. The positions of electrodes were verified by brain histology. For facilitating the identification of electrode array position, the electrode tips were dipped in fluorescent Neuro-DiI (Neuro-DiI, #60016, Red oily solid color, from Biotium Inc.), which can then reveal the electrode track. DAPI (4′,6-diamidino-2-phenylindole) staining was used for the counter staining of the nuclear DNA of brain cells. Images were collected using a Zeiss 780 Upright Confocal microscope.

### Recording in Wild-Type Mice and *Cre*/lox Transgenic Mice during Fearful Episodic Events

Mice were allowed to recover for approximately 1 week before experiments began. Following the recovery period, each connector-pin array was connected to pre-amplifiers from the Plexon system in tetrode format. Since our electrode contained 512 channels, we used four 128-channel Plexon systems synchronized by the Quad Controller feature. By using five helium balloons (each provided up to 49 g of airlift), the effect of the cable weight from the Plexon system and headstage (about a total of 225 g weight) can be dramatically reduced; so much so that the mouse is able to move again.

For optogenetic manipulation, we crossed CaMKII promoter-driven *Cre* mice (T62-Cre, Cui et al., 2004) with Arch mice for making the double transgenic mice (T62-cre::Arch). In these mice, we implanted our 512-channel electrodes that contained optrodes, which targeted both the S and AuV. The optical fibers were connected to a green laser (532 nm, diode-pumped solid-state, Shanghai Dream Laser Technology Co., Shanghai, China).

To examine neural firing changes in response to emotionally fearful events, we used two types of stimulation protocols: (1) A laboratory simulation of an earthquake (3000 rpm to shake a small 4″ × 4″chamber fixed on top of a vertex machine for 0.5 s (Lin et al., 2005; Oşan et al., 2011). To maintain the consistency of stimulus inputs, yet minimize the possible prediction of upcoming stimuli, the shake was triggered using a computer and delivered seven times at randomized intervals within a few minutes. (2) Fear-conditioning. The fear-conditioning chamber was a square chamber (10″ × 10″ × 15″, generated in the lab) with a 24-bar shock grid floor (Model E63-13, Coulborn Instruments, Holliston, MA, USA; Chen et al., 2009). Mice were placed into the shock chamber for 3 min and received the unconditioned stimulus (a continuous 285-ms foot-shock at 0.75 mA). In our experiments, we started the recordings 30 min before a series of earthquake or foot-shock episodes were delivered to the mice. Each given type of fearful events was delivered in a single session seven times with randomized time intervals ranging from 1 to 3 min (inter-trial intervals). A single earthquake session lasted for about 20 min, and the mice were then brought back to the home cages for a brief 5- to 10-min rest. This was followed by a fear-conditioning foot-shock session, consisting of seven randomized foot-shocks. The recordings continued for another 30 min post-events in the home cages.

### Spike Sorting and Data Processing

The neuronal activity was recorded by a Plexon multi-channel acquisition processor system (Lin et al., 2006a; Kuang et al., 2010), and waveforms were collected using 56 points with 1400 μs time width. The recorded spike activities from various brain regions were processed in the manner also previously described (Zhang et al., 2013; Li et al., 2015). Briefly, the spike activity was recorded using the Plexon Systems, then sorted using the MClust 3.3 program^1^.

First, the recorded data were filed in Plexon system format (^∗^.plx). Before spike sorting, the artifact waveforms were removed and the spike waveform minima were aligned using the Oﬄine Sorter 2.8 software^2^ (Dallas, TX, USA). The aligned data were then saved as files in a Neuralynx System format (^∗^.nst). Next, the MClust 3.3 program was used to isolate different spiking units. Only units with less than 0.5% in spike intervals within a 1-ms refractory period and clear boundaries were used. The well-separated neurons were assessed by “isolation–distance” and “*L*-ratio.” In the present analysis, neurons whose isolation–distance > 15 and *L*-ratio < 0.7 were selected for peri-event spike histogram and raster analysis (∼79.5% met this criteria). The stability of the ensemble recordings was judged by comparing waveforms and interspike intervals at the beginning, during, and after the experiments.

## Results

### Design and Construction of the 512-Channel 13 Site-Ensemble Recording Device

**FIGURE 1:**
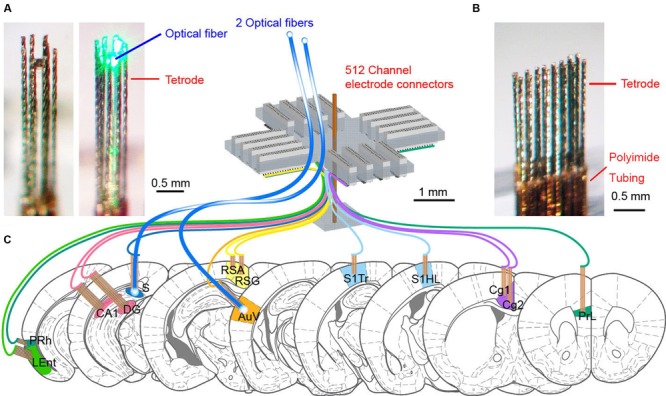
**The design of 512-channel tetrode arrays for brain-wide neural recording and optogenetic probing in mice. (A)** An eight-tetrode bundle with optical fiber in the center. The 3 × 3 array square formation was used to arrange eight tetrodes at the outline, surrounding the optical fiber at the center. The left one shows the optrode bundle with green laser-light off, whereas the right photo shows the optrode bundle with green laser-light on. **(B)** An example of a tetrode array consisting of two rows of eight evenly spaced tetrodes. The tetrode was cut into a specific length depending on the recording depth. The polyimide tubing used for holding the wires together remained above the cortical surface. **(C)** Schematic drawing for the 512-channel tetrode device for recording in a total of 13 different brain regions in mice. Two tetrode arrays targeting the subiculum (S) and secondary auditory cortex (AuV) also contained optical fibers for opsin-based manipulation of neural activity. The scales are marked by black bars.

Since tetrodes offer a superior spike-sorting capacity, we designed and constructed the 512-channel headstage system that holds 128 tetrodes as well as two optrodes (**Figure 1**). We selected 13 distinct brain regions based on their roles in the processing of fear-conditioning memory. These brain regions are prelimbic cortex (PrL), Cg1 and Cg2 subregions of the anterior cingulate cortices (ACC), retrosplenial agranular cortex (RSA), retrosplenial granular cortex (RSG), somatosensory cortex hind limb (S1HL), somatosensory cortex trunk (S1Tr), secondary auditory cortex (AuV), hippocampal CA1, dentate gyrus (DG), subiculum (S), lateral entorhinal cortex (LEnt), and perirhinal cortex (PRh). To record neuronal activities in these 13 distinct brain regions simultaneously, we employed a modular design to arrange our electrodes and optical fibers (**Figures 1A,B**). A total of 13 modular bundles of tetrodes was used to target these structures (**Figure 1C**).

**FIGURE 2:**
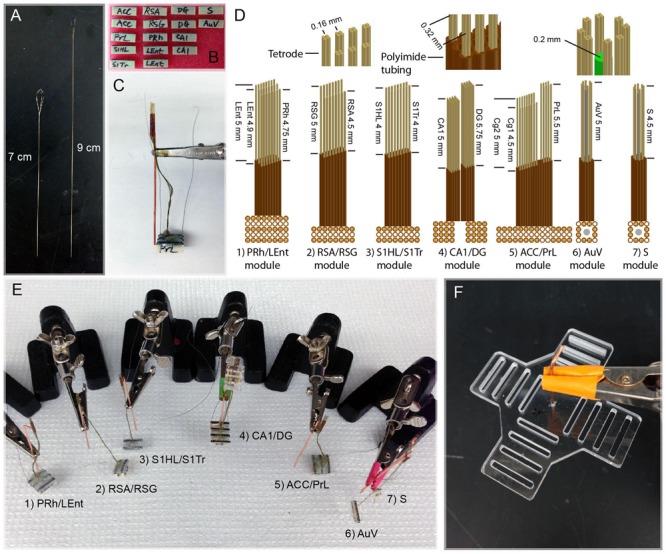
**Construction and assembly of 512-channel tetrodes and headstage. (A)** Two different lengths of tetrode wires. **(B)** A total of sixteen 32-channel connector pins were used to make a 512-channel headstage. **(C)** An assembled PrL/ACC (Cg1 and Cg2) module consisting of three 32-connector pins with 96-channel arrays (see the detailed arrangement in C:5, ACC/PrL module). The copper bar was used for temporally holding the assembled polyimide tubes and connector pins during the construction of the module and surgery. The tips of tetrodes protruding from the polyimide tubes are inserted into the brain. Two grounding wires were used for each module; one is to be attached to the skull and the other to the copper-mesh cage wrapped around the finished headstage after surgery. **(D)** Specifications of various tetrode arrays used for constructing seven different brain region modules. **(E)** Seven assembled tetrode array modules prior to surgical implantation. **(F)** The planar fiberglass used to secure four 128-channel connector pins (each consisting of four rows of 36 pin-connector arrays) on each arm. The copper bar in the middle of the fiberglass was used as a pole for holding the headstage to the dental-cement base in the skull and also for tethering to a helium balloon.

Because of the different depths of these brain structures, as well as the variable distances for the fine wires that need to be attached to the connector pins, we made these tetrodes two different lengths (7 cm vs. 9 cm; **Figure 2A**). This ensured that tetrodes had sufficient length to be inserted into the brain. The polyimide tubes holding the electrodes always stayed above the brain surface, thereby minimizing tissue damage. We found that extending the wires from 7 cm in length to 9 cm did not cause any noticeable loss in signal-to-noise ratio. Based on the size of each target area, we used either 32 or 64 channels and corresponding numbers of connecting pins (**Figure 2B**). For example, 64 channels were used in the two subregions of the ACC, with the first 32 channels designated for the ACC-Cg1 and the second 32 channels for the ACC-Cg2 (**Figure 2B**). Similarly, we used 64-channel modules for the two subregions of the retrosplenial cortices, with 32 channels for the RSA subregion, and 32 channels for the RSG subregion, respectively. Sixty-four channels were also used for the two subregions of the somatosensory cortex (32 channels for S1HL and 32 channels for the S1Tr) as well as for recording in the CA1, DG, and LEnt. On the other hand, we designated 32-channel tetrodes for the PrL, PRh, S, and AuV (**Figure 2B**). A reference wire was then soldered to each end of the 32-channel connector used in each module. The completed connector-pin array was sealed by epoxy for protection. The looped tetrodes were then glued to the base of the microdrive or immobile module.

For chronic recordings from these 13 brain regions, we minimized the number of holes on the mouse’s skull. We achieved this by grouping two or three nearby intended regions by a single module of tetrode arrays (**Figure 2C**). As such, a total of seven separate modules were constructed. Based on the unique anatomical shapes of each brain structure, we designed different configurations of tetrode arrays (**Figure 2D**). Within each module, segments of polyimide tubing were arranged in arrays that contained one or multiple bundles, which allowed one or multiple nearby brain sites to be targeted, respectively. These seven modules were constructed and shown (**Figure 2E**) as follows: (1) PRh/LEnt-module; (2) RSA/RSG-module; (3) S1HL/S1Tr**-**module; (4) CA1/DG-module; (5) ACC (Cg1/Cg2)/PrL-module; (6) AuV-module; and (7) subiculum (S)-module.

Depending on the number of channels for each brain region and the shape of these regions, the tetrode arrays in each module and their arrangement varied accordingly. For example, the LEnt/PRh module consisted of three arrays of polyimide tubes (with each tube holding one tetrode). Eight tubes in a row were used for the PRh, and two rows of eight tubes were used for targeting the LEnt. Thus, this PRh/LEnt module utilized a total of 96 channels. The three tetrode rows within this module were separated by two empty rows of polyimide tubes as shown (**Figure 2D**). Moreover, due to the different depths of recording sites in the LEnt and PRh, we cut the tetrodes to the different lengths. This similar process was used to build other individual modules – such as RSA/RSG-, S1HL/S1Tr-, and ACC/PrL-modules.

Because the CA1 and DG regions are only one or two cell-layer thick, it requires fine adjustment of the position of the tetrodes to increase the chance for maximal recordings from these principal neurons. Accordingly, the polyimide tubes that were to be inserted in the CA1 and DG were affixed to moveable-screw nuts on a microdrive base constructed from fiberglass.

For the modules used for recordings in the S or the AuV, we also incorporated the optrode design, consisting of an optical fiber surrounded by eight tetrodes. In our optogenetic stimulation, we used 200 μm core, 037NA standard cladding multimodal optical fiber. After the cladding was removed, a bundle of 12 polyimide tubes was arranged surrounding the fiber (see the upper and lower sub-drawings on the right side of **Figure 2D**).

In total, 16 connector-arrays were used for each 512-channel headstage, and these seven modules were constructed and assembled in a stepwise manner (**Figure 2E**). To hold various tetrode modules and balance the weight of the 512-channel headstage, we used a cross-shaped planar arrangement with four 128-channel modules (each consisting of four rows of 36 pin-connector arrays) positioned in each arm (**Figure 2F**). Based on our experiences, a 512-channel electrode headstage can be built by a skilled technician in five days or less.

**FIGURE 3:**
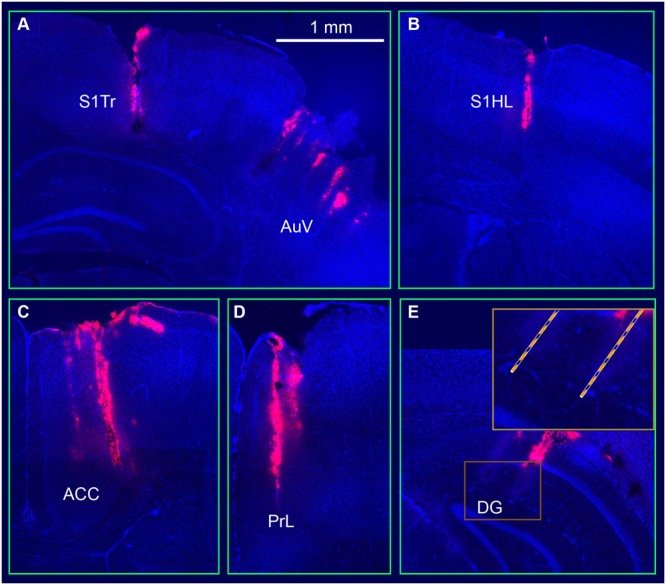
**Visualization and verification of representative tetrode tracks in the mouse brain. (A)** Tetrode arrays in the somatosensory cortex (S1Tr) and the auditory cortex (AuV). **(B)** A tetrode track in the S1HL. **(C)** A tetrode track in the prefrontal cortex (ACC). **(D)** A tetrode track in the prelimbic cortex (PrL). **(E)** Two tetrode tracks in the hippocampal dentate gyrus (DG). The square box is enlarged at a higher magnification.

Once the above tetrode modules were constructed, we used the sequential-insertion strategy to implant the 512-channel system into the adult male mice (4- to 6-month old with a body weight of 35–40 g). In the present study, we used five such headstages and implanted in three wild-type mice and two CaMKII-Cre (T62)::Arch double transgenic mice. During surgery, seven small holes were drilled in the mouse skull based on their corresponding coordinates. We first inserted the RSA- and RSG-tetrode module, followed by the S1Tr- and S1HL-module. Next, the ACC and PrL module was implanted. The optrode’s modules, targeting the S and AuV, were the next inserted, respectively. The CA1 and DG modules, which were attached to a fiberglass microdrive, were implanted before the final module targeting the PRh and LEnt was inserted. Using this sequential strategy, we were able to complete the surgery in about five hours for each mouse. The electrode positions were verified after all of the chronic recording experiments were completed. To facilitate the identification of electrode array positions, the electrode tips were dipped in fluorescent Neuro-Dil, which can then reveal the electrode tracks and their positions in the intended target area (**Figure 3**).

### Network Dynamics among the 13 Brain Structures

**FIGURE 4:**
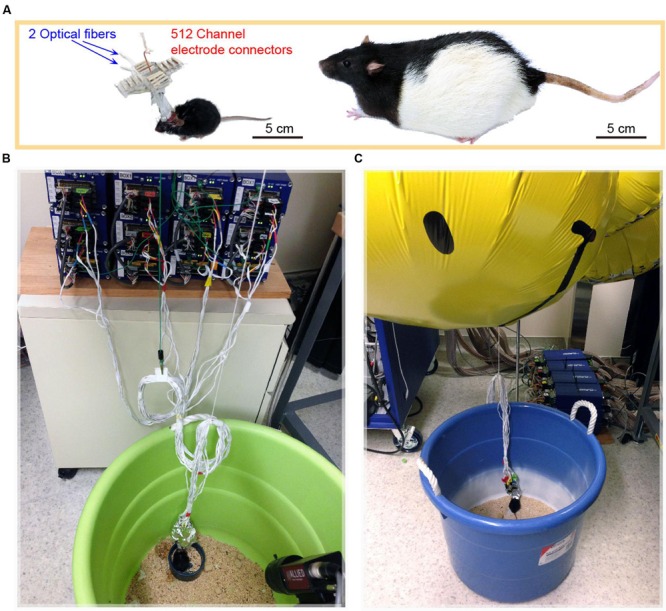
**Mice implanted with 512-channel headstage for recording from 13 brain structures. (A)** A mouse with a 512-channel headstage, which also contained two optical fibers for light stimulation of the AuV and subiculum (S), respectively. The mouse is an order of magnitude smaller than a Long Evans rat which is typically used for *in vivo* neural recording. **(B)** The 512-channel headstage was connected through the ultra-thin cables to the 512-channel Plexon Neural Data Acquisition System. **(C)** A helium balloon was used to alleviate the weight of cables and headstage, enabling the mouse to perform behavioral tasks.

**FIGURE 5:**
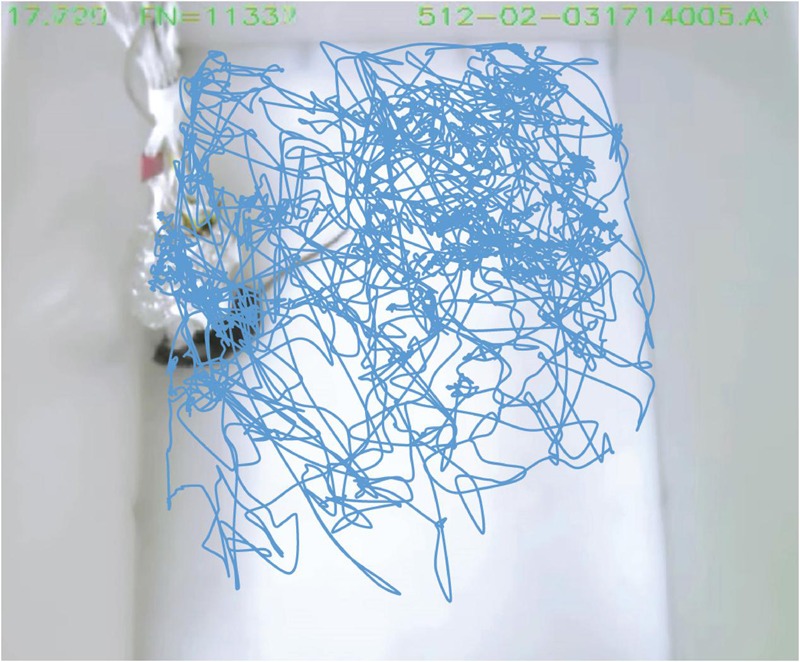
**Mice implanted with the 512-channel headstage can still move freely during behavioral tests.** The video track shows the movement trajectories inside a novel chamber during behavioral exploration. A total of 10 min were plotted. The object contour tracking mode in CinePlex Studio Application Version 3.2 (Plexon Inc.) was used to track the movement trajectories.

Despite the fact that the size of mice is only one tenth that of rats (**Figure 4A**), the mouse with the 512-channel headstage (which weight about 8.6 g) could still move around well enough to obtain food pellets and water. However, once the 512-channel headstage was linked with the pre-amplifiers and cables (**Figure 4B**), a significant amount of weight was added (about a total of 225 g) that severely constrained the mouse’s movement. To solve the cables’ weight issue, we used five large helium balloons (from Walmart, each balloon provides 49 g weight lift) tethered to the cables to relieve its weight (**Figure 4C**). We further adjusted the amount of airlift by adding or reducing the number of small metal screw rings tied to the bottom of the balloons. After acclimation, the use of helium balloons enabled the mice to move again and perform behavioral tasks [see the plotted movement trajectories during the exploration of a novel environment (**Figure 5**)].

**FIGURE 6:**
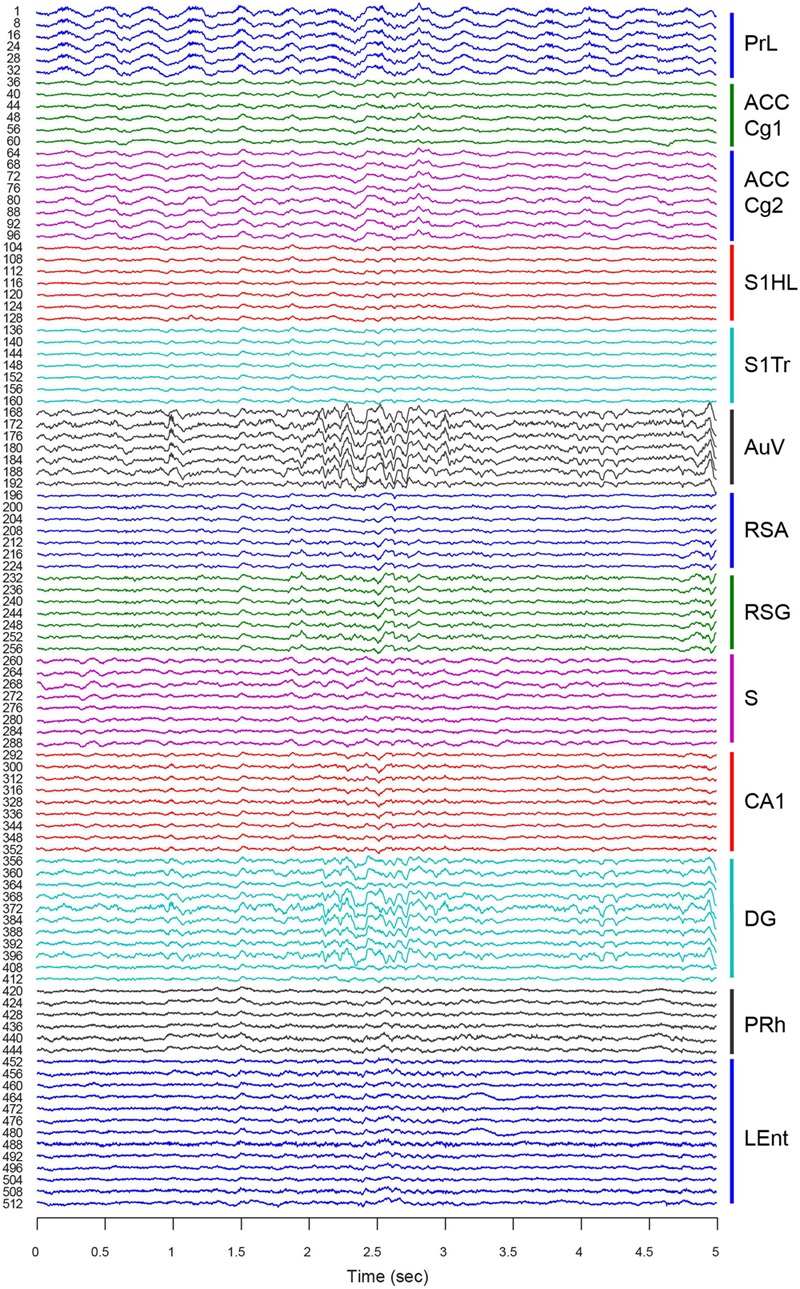
**Large-scale local field potentials (LFP) simultaneously recorded from 13 different brain regions.** An LFP signal was collected from one out of every four channels for each tetrode. This provided a total of 128 channels of LFP signals. A 5-s segment of LFP is used as an example. LFPs from different tetrodes within each region are more similar than those of different regions. Please note LFP in the AuV seemed to be more coherent with LFP in the DG (see large oscillatory signals at the time period from 2.1 to 2.7 s). Thirteen brain regions are labeled on the right with different colors.

For chronic recordings, we typically allowed the mice that underwent surgery to recover for 3∼5 days before experiments began. Neural-spike activities and LFPs were recorded once the 512-channel cables were connected to the 512-channel Plexon multiplex-recording system. We verified the electrode connections using LFPs. Since LFP tends to be similar to nearby tetrode channels, we sampled one out of every four channels for LFP. Therefore, this 512-channel LFP-recording setup provided a total of 128 channels for LFP signals. As expected, the overall network oscillation among the local electrode sites within each brain structure was quite similar (**Figure 6**, e.g., ACC Cg1 vs. ACC Cg2, S1HL vs. S1Tr, and RSA vs. RSG). Also, the regions next to each other (with direct connections) tended to oscillate coherently (e.g., the PrL vs. ACC, or PRh vs. LEnt). However, from the visual inspection, we noted that the LFP from the AuV was rather similar to that of the DG, suggesting more direct-interactions between these two regions at that particular moment in time.

**FIGURE 7:**
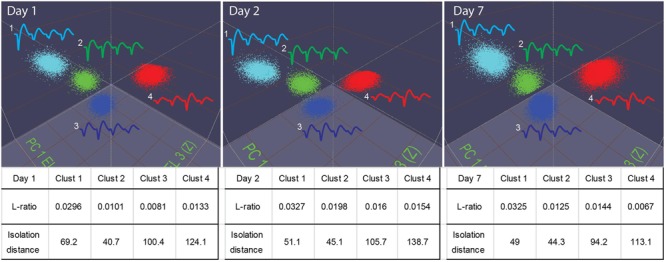
**Stable recordings of single units in mice.** The top three panels show four well-isolated units in the PCA plot used in the MClust recorded from the same tetrode. These units remained stable over a period of a week (from Day 1 to Day 7). The *L*-ratio and isolation distance corresponding to these four units are listed beneath the panel, the table provides quantitative measures for the separation quality for these units.

Our 512-channel system can hold electrodes in position for many days. Stable recordings were verified by the PCA-based spike-sorting pattern obtained from the same electrodes (**Figure 7**). As judged by “isolation–distance” and “*L*-ratio”, many units recorded (e.g., see examples from the RSG) maintained good separation and stability over days and week(s).

### Single-Unit Responses across Different Brain Structure upon Fearful Stimulation

It has been widely assumed that memories are distributed across many brain regions (Lashley, 1950, 1963). However, traditional *in vivo* recordings were typically limited to one or two sites at a time. To demonstrate the feasibility of large-scale and multi-structure recording in the mouse brain during memory formation, we subjected the mice to two distinct types of fearful episodes namely, laboratory-simulation of earthquake and fear-conditioning foot-shock. We have previously shown that earthquake-like events were fearful to mice and can trigger changes in heart rates and heart rate variability similar to those of fear-conditioning (Liu et al., 2013, 2014). Moreover, earthquake-like stimuli induced diverse changes in the CA1 region of the mouse hippocampus (Lin et al., 2005, 2006b; Oşan et al., 2011). In the present study, we ask an earthquake and foot-shock evoke firing changes in a brain-wide manner.

**FIGURE 8:**
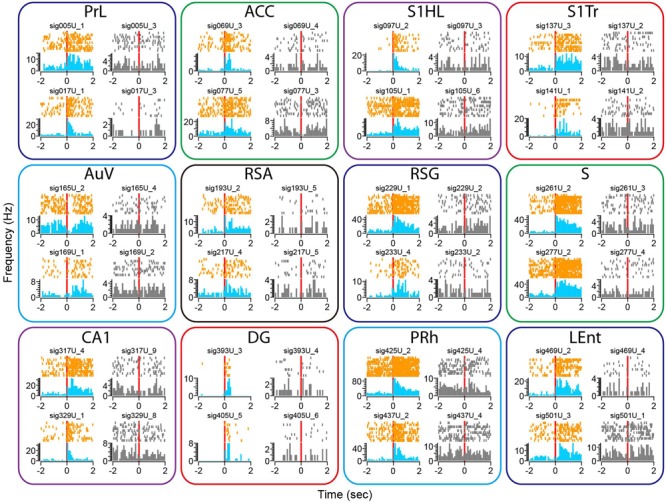
**Earthquake event triggered robust changes in firing rates in some of the recorded units across all 13 brain regions.** Peri-event spike raster and histograms illustrated two example units recorded from the same tetrode from each of the 13 brain regions, placed in top and bottom rows. The left side of subplots shows the units with firing changes (peri-event spike raster in orange color, whereas peri-event spike histogram in light blue), the right side of subplots showing another representative unit (in gray) recorded from the same tetrode did not show significant firing change. Please note that only units of the Cg1 (but not Cg2) of the ACC are shown here.

We subjected the mice to an earthquake seven times and monitored how neurons responded. Using peri-event spike raster and histogram analysis, we found that earthquakes (0.5 s in duration) triggered robust firing changes in a subset of the recorded neurons across all of the 13 brain structures (**Figure 8**). This robust change in firing was not due to a mechanical artifact, because many single units recorded from the same tetrode showed stable firing rates (these unresponsive units were shown in gray, whereas the responsive units from the same tetrodes were plotted in orange color for peri-event raster and in blue for peri-event spike histogram).

There were also a variety of response dynamics in the brain regions. For example, some units exhibited increased firing rates (i.e., PrL), while others showed a transient reduction in firing (i.e., the first top units in the AuV). Likewise, some units had transient increases that seemed to be time-locked to the stimulation *per se* (i.e., two units from the DG), whereas other units showed prolonged firing increases (i.e., units in the S). In addition, both the putative excitatory units and putative interneurons exhibited firing changes (i.e., the top unit in the PRh as a putative interneuron vs. the top unit from the S1HL as a putative pyramidal cell).

**FIGURE 9:**
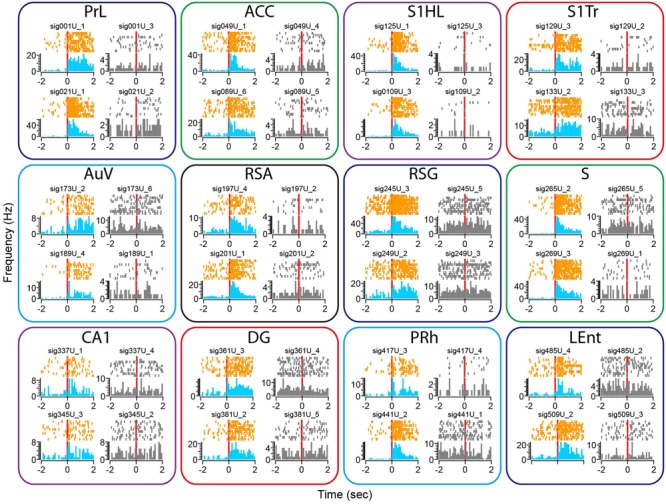
**Foot-shock triggered robust changes in firing rates in some of the recorded units across all 13 brain regions.** Peri-event spike raster and histograms illustrated two example units recorded from the same tetrode from each of the 13 brain regions, placed in top and bottom rows. The left side of subplots shows the units with firing changes (peri-event spike raster in orange color, whereas peri-event spike histogram in light blue), the right side of subplots showing another representative unit (in gray) from the same tetrode did not show significant firing change. Please note that only units in the Cg1 of the ACC are shown here.

To examine whether our 512-channel recording headstage can be used for the study of a fear-conditioning paradigm, we subjected the mice to classic mild electric foot-shock from a 24-bar shock grid floor (Chen et al., 2009; Zhang et al., 2013). Mice were placed into the shock chamber for 3 min and then received the unconditioned stimulus (a continuous 285-ms foot-shock at 0.75 mA) seven times at 1- to 3-min time intervals. Although electric noise from the electric foot-shock was noticed in the Plexon recording system, it can be easily removed from neural signals upon spike sorting. Similar to the widely distributed neural responses to earthquake, fear-conditioning foot-shock also evoked diverse changes in single-unit firing rates across all 13 brain regions (**Figure 9**). We found most of the responsive neurons exhibited prolonged increases in spike discharge, lasting over many seconds. This also demonstrated that their firing changes were not due to contamination of electric noise from the electric shock, which lasted only 0.287 s. Moreover, from the same tetrode we can identify specific units showing foot-shock-induced firing changes (subplots in color), whereas many other cells remained unchanged (subplots in gray).

### *Cre*/lox-Mediated Region-Specific Optogenetic Probing of Cross-Structure LFP Interactions

Because *Cre*/loxP neurogenetics and opsin-based optogenetics are important tools for investigating how specific genes and/or specific cell types contribute to network dynamics, we then incorporated optical fibers into our 512-channel recording arrays. Here, we implanted the optrodes in the double transgenic (CaMKII::Cre/Arch) mice in which we can selectively suppress neural activity of CaMKII-positive principal neurons by green light. We selected two brain structures for simultaneously placing the optical fibers – namely, the secondary auditory cortex (AuV) and subiculum (S). Each optrode contained one optical fiber in the center and eight tetrodes arranged in a square (with two tetrodes on each of the four sides, **Figure 2D**, the upper right drawing, as well as the lower right drawings, labeled AuV and S).

While any site can be tested for optrodes, we selected these two sites based on consideration from fear conditioning and fear-memory consolidation perspective. It is known that the AuV serves as a cortical relay station as the auditory information from the primary sensory cortex is broadcasted into the limbic and other neocortical structures, whereas the subiculum is positioned as a critical outpost for broadcasting the hippocampal information back to the cortical areas. By selecting one cortical and one limbic structure, we can ask how the population-level dynamics across a wide range of brain structure might be differentially influenced by the artificial suppression of principal cells in specific circuits.

**FIGURE 10:**
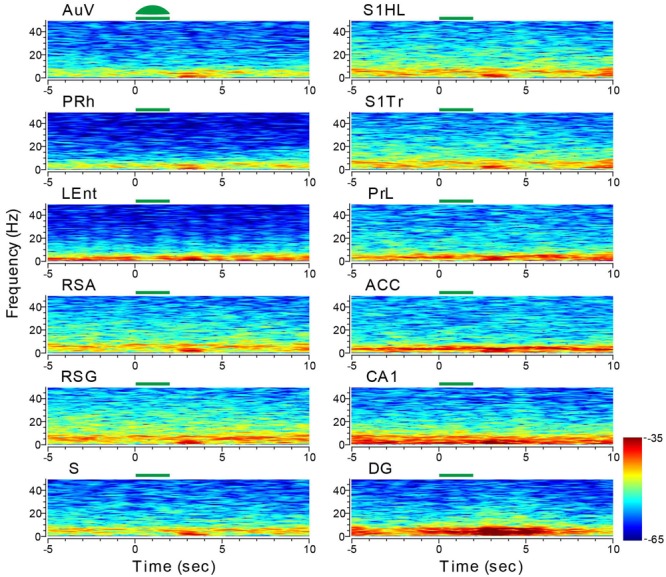
**Optogenetic stimulation in the auditory cortex triggered dynamic changes in LFP across the 13 brain regions.** Green-laser light stimulation (10 Hz) in the AuV of the CaMKII-*Cre*/Arch transgenic mice showed suppressed neural oscillation at lower frequencies during the 2-s light-stimulation, but caused apparent rebound theta oscillation across nearly all 13 brain areas after the termination of light stimulation at the AuV. Green bars at the top of the LFP power spectrum indicate the light stimulation duration. LFP color maps were averaged over 10 trials.

We first stimulated the AuV using 10-Hz stimulations for 2 s (10 ms per pulse of green laser at 10 mW, 20 pulses per train) in 10 trials. This produced a noticeable reduction of LFPs in a 10-Hz range (**Figure 10**). This reduction was also noted in other areas, such as the LEnt and subiculum. Interestingly, we observed a dramatic rebound and overshoot in the 10-Hz LFP oscillation at the termination of the 2-s light stimulation (**Figure 10**). This rebound LFP oscillation was observed across all of the 13 recorded sites with a short latency of ∼0.5 s or less, and was typically ∼2 s in duration with the DG exhibiting the longest (∼4 s) rebound excitation.

**FIGURE 11:**
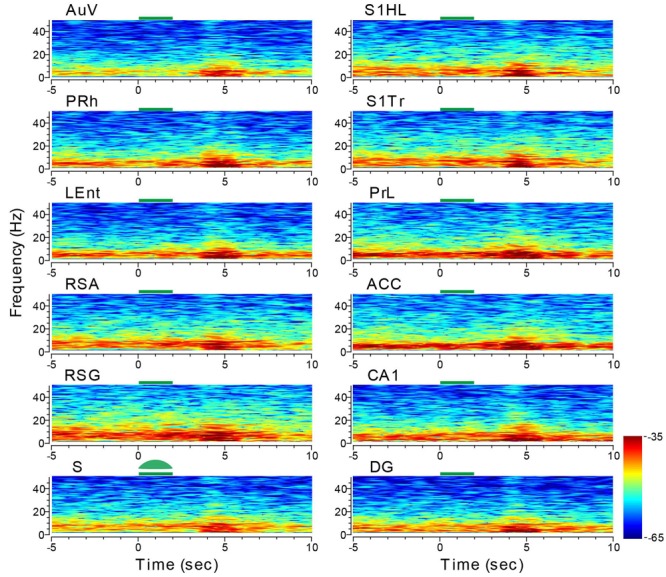
**Optogenetic stimulation in the subiculum triggered dynamic changes in LFP across the 13 brain regions.** Green-laser light stimulation (10 Hz) in the subiculum (S) of the CaMKII-*Cre*/Arch transgenic mice showed suppressed neural oscillation at lower frequencies during the 2-s light stimulation, but caused strong rebound theta oscillation across nearly all 13 brain areas after the termination of light stimulation at the subiculum. Green bars at the top of the LFP power spectrum indicate the 2-s light stimulation duration in the subiculum. LFP color maps were averaged over 10 trials.

We then stimulated the subiculum with the green laser light using the same stimulus parameters. While the reduction in LFP amplitude during the light-stimulation was not obvious, the delayed rebound in oscillation intensity (i.e., theta range and low gamma, etc.) was clearly evident – not only in the subiculum, but also across all other recorded structures (e.g., the adjacent sites such as the LEnt and PRh, DG showing large changes in amplitude, **Figure 11**). The latencies between the termination of the subiculum light suppression and the onset of rebound theta in most brain regions were ∼1.5–2 s in duration. This brain-wide response to a specific light suppression in a given subregion is also consistent with the notion that site-specific optical manipulation produces unexpected changes in network dynamics and patterns across the whole brain.

### Optogenetic Manipulation Across Different Brain Structure

**FIGURE 12:**
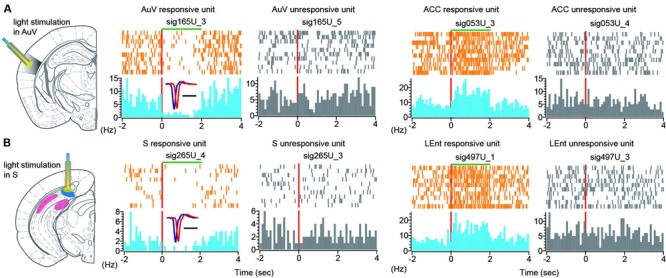
**Single-unit responses to green light stimulation. (A)** Peri-event spike raster (in orange color) and spike histogram (in light blue) show that a responsive unit in the AuV suppressed its firing in time-locked manner during 2-s light stimulation (left spike raster). Another unit recorded from the same tetrode did not change its firing (second left panel, in gray). Some neurons in the other brain regions also responded to the AuV light stimulation. Two units from the ACC were plotted, one unit exhibited increased firing changes (in color), and another unit (in gray) from the same tetrode did not change its firing rate. **(B)** Peri-event spike raster and histogram show that a unit in the subiculum reduced its firing during 2-s light stimulation (left spike rasters in color). Another unit recorded from the same tetrode did not change its firing (in gray). Two units from the LEnt were plotted here, one unit exhibited increased firing changes (in color), and another unit from the same tetrode did not change its firing (in gray).

To further investigate the effects of optogenetic suppression at the single-neuron level, we performed peri-event spike histogram analysis on the spike datasets. Indeed, we found that some units in the AuV decreased firing in response to the 2-s green light stimulation (**Figure 12A**, the unit labeled as sig165U_3 in the first left subpanel in color). This decreased firing was time-locked to the duration of light stimulation, while other units (labeled as sig165U_5) simultaneously recorded from the same tetrode did not alter their firing (**Figure 12A**, the second subpanel from left in gray), suggesting that this optogenetic suppression effect was specific.

Interestingly, we also found that some units in other brain regions exhibited altered firing in response to green light stimulation. For example, an ACC unit (sig053U_3) showed increased firing time-locked to the 2-s duration of green light stimulation (**Figure 12A**, right side of two subpanels), whereas another ACC unit (sig053U_4) from the same tetrode did not alter its firing. Again, this ACC unit exhibited time-locked increase to the 2-s light stimulation duration in the AuV.

Similarly, turning on the green light in the subiculum was also effective in suppressing its cells’ firing (**Figure 12B**, left subpanels showing the unit sig265U_4 in orange and blue color). Furthermore, the firing suppression was specific, whereas other units recorded from the same tetrode did not change their firing (**Figure 12B**, left subpanel in gray showing the unit sig265U_3). We also observed that some units in other brain regions also responded to the green light-stimulation of the subiculum. For example, one unit (labeled as sig497U_1) in the LEnt responded to the 2-s subiculum light stimulation with elevated firing for the stimulus duration of the green light. Another unit (labeled as sig497U_3) from the same tetrode was not responsive to the subiculum light stimulation. Taken together, these results demonstrated that optrodes worked well in our 512-channel recording headstage. Moreover, light stimulation at one brain site triggered dynamic changes in both LFP patterns and single-unit firing dynamics across many brain regions.

## Discussion

To our knowledge, the device we have described here is the first 512-channel tetrode headstage that has allowed researchers to record single units and LFPs from 13 different brain regions simultaneously in behaving mice. The two major advantages of our microelectrodes are the low-cost and the do-it-yourself versatility. Silicon probes, which can offer large channel numbers for large-scale recordings in primates (such as the 10 × 10 Utah array), are typically manufactured by commercial companies. A Utah array for 10 × 10 electrodes costs ∼$5000. These standard arrays contain a single electrode with a fixed probe length, often limiting recordings to the cortex of large animals. Another popular probe is the Michigan Probe offered by NeuroNexus. But the channel numbers for the probes used for recordings in rodents are usually much smaller – say, between 16 and 32 channels (Okun et al., 2016). Recently, a customized large-scale silicon probe array was recently reported for recordings in rats (Berényi et al., 2014; Buzsáki et al., 2015), and showed impressive LFP recordings from many sites within the rat hippocampus. At the time of this writing, a standard matrix probe (fixed configuration) for chronic rat recordings with 64 channels or 128 channels is $2370 or $3405 (other parts not included). These standard probes also do not come with optrodes. According to the NeuroNexus website, the company offers a custom probe design service with a minimum order for a standard 16∼32 channel probe of $7500, or premium probes with >64 or more channels of $20,000 (the estimated cost per probe is unknown, because it is dependent on design)^3^. This means that many laboratories that are not as well-funded would likely be unable to afford such high-count arrays or to explore various designs necessary for optimizing probe configurations.

By comparison, our 512-channel microwire probes can be made within laboratories at a fraction of the cost (see Supplementary Tables S1 and S2 for the items and tools required for assembling 512-channel electrodes). Importantly, this do-it-yourself procedure can enable researchers to readily modify the channel numbers and electrode depths to target any set of brain structures. For example, we used adjustable microdrives for targeting the principal cell layers of the DG and CA1 of the mouse hippocampus. Depending on the unique need of each experiment, any of the electrode bundles can be made adjustable to further increase the unit yield. Of course, the microdrive comes at a price by having increased weight and more moving parts.

In addition, these 512-channel microelectrodes can be readily equipped with optical fibers, allowing recordings and optogenetic manipulation experiments for various projects within laboratories. Using the present configuration, we monitored neural activity patterns across 13 brain regions and found that natural complex stimuli, such as earthquake or foot-shock, triggered robust changes in both single-unit activity and LFP. This observation is consistent with the speculation that the neural code representing episodic experiences is distributed across the brain.

Finally, one surprising finding from the 13-region simultaneous recordings is that the optogenetic suppression of a given region (i.e., whether it was in the subiculum or auditory cortex) strongly altered ongoing LFP and caused a delayed rebound oscillation at theta frequency in all of the brain regions examined. This abnormal rebound in theta range may be directly related to the 10-Hz green light stimulation frequency. We believe that it is unlikely that this rebound was an artifact because of the different time latencies triggered by AuV optical stimulations vs. subiculum optical stimulations. Although we haven’t done extensive control experiments for the present 512-channel methodology study, our pilot set of optogenetic stimulations in the cortical or subcortical areas in the DAT-*Cre*/ChR or PV-*Cre*/ChR lines did not produce such a delayed rebound phenomenon (data not shown). This also excluded the possibility that such a rebound was simply due to temperature changes induced by 2-s laser illumination. In addition, our laser stimulation was delivered when the mice were in a quiet awake state, we did not notice any overt locomotion upon laser stimulation. This would also go against the argument that such changes were due to the sudden appearance of a green light in the recording environment (the headstage and optical fiber were completely wrapped in aluminum foil (see **Figure 4B**). In consideration of the above conditions, we would like to suggest that the rebound was related to specific CaMKII-positive neural types/pathways rather than the artifacts of laser illumination. If this is the case, our findings may raise a potentially cautionary note on the interpretation of some of the optogenetic manipulation experiments, which reported altered memory functions, especially when freezing behavior was used as the index for measuring memory (i.e., Liu et al., 2013; Ramirez et al., 2015; Roy et al., 2016). In this regard, it will be of interest to further characterize this rebound phenomenon in future experiments.

In summary, we described a 512-channel and 13-brain-region neural recording technique for mapping and manipulating neural activity in the brain of freely behaving mice. This 512-channel device has the major advantages in its proven reliability and low-cost. It can be readily constructed within laboratories for targeting any set of brain sites of interest, and may further expand to an even larger channel count. However, it should be noted that one major bottleneck facing such super-scale recordings is the cables and size of the connecting pins. These issues need to be addressed in the future.

## Author Contributions

All authors listed have made substantial, direct and intellectual contribution to the work, and they have approved it for publication.

## Conflict of Interest Statement

The authors declare that the research was conducted in the absence of any commercial or financial relationships that could be construed as a potential conflict of interest.

## Footnotes

1 http://redishlab.neuroscience.umn.edu/MClust/MClust.html

2 http://www.plexon.com

3 http://neuronexus.com/products/custom-designs/custom-probe-design
