# Supplementary material for: 512-Channel and 13-Region Simultaneous Recordings Coupled with Optogenetic Manipulation in Freely Behaving Mice
Source: Front Syst Neurosci. 2016 Jun 14;10:48. doi: 10.3389/fnsys.2016.00048 (PMC4905953; doi:10.3389/fnsys.2016.00048)
Supplement: Supplementary file 1 [file Table_1.DOCX]

Table 1 Construction of 512-channel headstage

|  | **Item** | **Vendor** | **Cost** |
| --- | --- | --- | --- |
| 1 | Polyimide tubing (2000020-50M) (TSP100170) | Polymicro Technologies, Phoenix, AZ | $300.00/50 meters |
| 2 | Optically clear and colored cast acrylic (8560K177) | McMaster-Carr, Douglasville, GA | $43.29/each |
| 3 | Stranded fixture wire (7715T11) | McMaster-Carr, Douglasville, GA | $0.13/ft. |
| 4 | Easy-to-form 260 brass rod (88605K43) | McMaster-Carr, Douglasville, GA | $2.46/pkg. |
| 5 | 200µm core, 037NA standard cladding multimode fiber (FT200EMT) | ThorLabs, Newton, NJ | $1.50/meter |
| 6 | Compact power and energy meter console, digital 4" LCD (PM100D) | ThorLabs, Newton, NJ | $1030.00/each |
| 7 | Zen toolworks CNC DIY kit 12x12 (F8 version) | ZEN Toolworks, Walnut Creek, CA | $679.99/each |
| 8 | 0.001 Platinum 10% Iridium (CFW Material #: 100-167), annealed temper, insulated with HML-Natural and a VG Bondcoat, Spooled (cfw-101), round wire | California Fine Wire Company, Grover Beach, CA | $0.60/ft. |
| 9 | Variable temperature heat gun (8977020) | Milwaukee, Brookfield, WI | $159.64/each |
| 10 | Electrode impedance tester (model IMP-1) | Bak Electronics, Umatilla, FL | No longer available |
| 11 | NPD-36-VV-GS, 36 position dual row male nano-miniature (.025"/.64mm) connector (A79026-001) | Omnetics, Minneapolis, MN | $27.97/each |
| 12 | Power adjustable option for SDL-473-200MFL | Shanghai Dream Laser Technology Co., Ltd., Shanghai, China | $240.00/each |
| 13 | 200mW 473nm MM fiber coupled laser (SDL-473-200MFL) | Shanghai Dream Laser Technology Co., Ltd., Shanghai, China | $2700.00/each |
| 14 | Vannas spring scissors - 3mm cutting edge (15000-00) | Fine Science Tools, Foster City, CA | $327.00/each |
| 15 | Dumont #5 forceps (11251-30) | Fine Science Tools, Foster City, CA | $33.00/each |
| 16 | Dumont #4 forceps (11241-30) | Fine Science Tools, Foster City, CA | $32.75/each |
| 17 | Fine scissors - sharp (14060-10) | Fine Science Tools, Foster City, CA | $70.75/each |
| 18 | 5-minute epoxy gel (14240) | ITW Polymers Adhesives North America, Danvers, MA | $4.98/each |
| 19 | Loctite liquid super glue (LOC1710908) | STAPLES, Framingham, MA | $6.79/pack |

Table 2 Implantation of 512-channel headstage

|  | **Item** | **Vendor** | **Cost** |
| --- | --- | --- | --- |
| 1. | Screws (B002SG89S4, #00-90, 1/8 inches) | Amazon, Seattle, WA | $32.48/25 screws |
| 2. | Dental cement kit, 1lb, pink opaque (59458) | Stoelting, Wood Dale, IL | $75.00/each |
| 3. | Extra-thin bass perforated sheet (mesh) (9360T12) | McMaster-Carr, Douglasville, GA | $57.27/ft. |
